# Assessing lameness prevalence and associated risk factors in crossbred dairy cows across diverse management environments

**DOI:** 10.1186/s12917-024-04093-w

**Published:** 2024-05-25

**Authors:** Priyanka Patoliya, Mukund A. Kataktalware, Kathan Raval, Letha Devi G., Muniandy Sivaram, Selladurai Praveen, Priyanka Meena, Sakhtivel Jeyakumar, Anjumoni Mech, Kerekoppa P. Ramesha

**Affiliations:** 1 Southern Regional Station, ICAR- National Dairy Research Institute, Bengaluru, 560030 India; 2 ICAR-National Institute of Animal Nutrition and Physiology, Bengaluru, 560030 India

**Keywords:** Commercial farms, Dairy cows, Lameness, Risk factors, Prevalence, Smallholder dairy farms

## Abstract

**Background:**

A thorough understanding of lameness prevalence is essential for evaluating the impact of this condition on the dairy industry and assessing the effectiveness of preventive strategies designed to minimize its occurrence. Therefore, this cross-sectional study aimed to ascertain the prevalence of lameness and identify potential risk factors associated with lameness in Holstein Friesian crossbred cows across both commercial and smallholder dairy production systems in Bengaluru Rural District of Karnataka, India.

**Methods:**

The research encompassed six commercial dairy farms and 139 smallholder dairy farms, involving a total of 617 Holstein Friesian crossbred cattle. On-site surveys were conducted at the farms, employing a meticulously designed questionnaire. Lameness in dairy cattle was assessed subjectively using a locomotion scoring system. Both bivariate and binary logistic regression models were employed for risk assessment, while principal components analysis (PCA) was conducted to address the high dimensionality of the data and capture the underlying structure of the explanatory variables.

**Results:**

The overall lameness prevalence of 21.9% in commercial dairy farms and 4.6% in smallholder dairy farms. Various factors such as age, body weight, parity, body condition score (BCS), floor type, hock and knee injuries, animal hygiene, provision of hoof trimming, and the presence of hoof lesions were found to be significantly associated with lameness. Binary logistic regression analysis indicated that the odds of lameness in crossbred cows increased with higher parity, decreased BCS, presence of hard flooring, poor animal hygiene, and the existence of hoof lesions. These factors were identified as potential risk factors for lameness in dairy cows. Principal component analysis unveiled five components explaining 71.32% of the total variance in commercial farms and 61.21% in smallholder dairy farms. The extracted components demonstrated higher loadings of housing and management factors (such as hoof trimming and provision of footbath) and animal-level factors (including parity, age, and BCS) in relation to lameness in dairy cows.

**Conclusions:**

The findings suggest that principal component analysis effectively reduces the dimensionality of risk factors. Addressing these identified risk factors for lameness is crucial for the strategic management of lameness in dairy cows. Future research in India should investigate the effectiveness of management interventions targeted at the identified risk factors in preventing lameness in dairy cattle across diverse environments.

**Supplementary Information:**

The online version contains supplementary material available at 10.1186/s12917-024-04093-w.

## Introduction

Lameness poses a significant health challenge in Indian dairy animals and globally, characterized as a clinical disorder impacting the locomotor system and adversely affecting cow locomotion, posture, and overall mobility [1]. The repercussions of lameness extend to various financial losses, encompassing diminished milk production, body weight loss, compromised fertility, escalated handling costs for treatments and medications, and the involuntary culling of animals [2–4]. Additionally, lameness induces pain and suffering, thereby diminishing the overall welfare of dairy cows [5, 6]. The global prevalence of lameness exhibits considerable variability, with reported rates ranging from 9.1% in Ireland [7] to 29.7% in Germany [8], 26.6% in the United States of America [9] to 30.1% in the United Kingdom [10], and even as high as 42.5% in Brazil [11]. A global analysis revealed a mean prevalence of lameness at 22.8%, with herd prevalence ranging widely from 0 to 88% [12]. In India, the prevalence of lameness in dairy cows varies from 8.1 to 30.5% [13–17]. Notably, the significant variation among existing reports on lameness prevalence in Indian dairy cattle is likely attributed to differences in methodologies employed for lameness identification.

Lameness, being influenced by a complex interplay of factors, involves a multifactorial aetiology encompassing aspects related to housing, management practices, and specific characteristics of the animals. The understanding of these risk factors within specific geographical areas is crucial for effective lameness control strategies. Numerous researchers, both in India and elsewhere, have delved into exploring potential risk factors associated with lameness [17–19]. A systematic review identified a total of 128 factors linked to lameness [20]. Notable animal-related risk factors include a low body condition score, the presence of claw overgrowth, larger herd sizes, higher parities, and the early stage of lactation. In Indian breeds, additional factors such as animal hygiene and hock joint ulceration were recognized for their association with a high prevalence of lameness [17, 19]. Various housing factors, including stall characteristics, lying area dimensions, and configuration, have also been associated with the prevalence of lameness [19, 21–23]. The type of flooring surfaces can sometimes contribute to the occurrence of hock and carpal joint injuries, acting as risk factors for lameness in dairy cattle [17, 22, 24]. Additionally, access to pasture or loafing areas has been identified as a protective factor against lameness in confined dairy cows, suggesting that grassland has a beneficial impact on gait condition [21, 24, 25].

Comprehensive knowledge of lameness prevalence is pivotal for assessing the impact of this condition on the dairy industry and evaluating the effectiveness of preventive strategies aimed at reducing its occurrence. Additionally, it is crucial to understand and identify the risk factors associated with lameness prevention. Considering risk factors at the individual, herd, and farm levels is essential when selecting the most effective strategies for preventing lameness in dairy cows [5, 26–28]. Despite significant efforts in lameness prevention, concerns persist about the increasing prevalence of lameness in dairy herds. Therefore, the primary objective of this study was to determine the prevalence of lameness and explore the relationships between various risk factors and the prevalence of lameness in dairy cattle across diverse commercial and smallholder dairy farms.

## Materials and methods

### Data collection

The research encompassed six commercial dairy farms (*n* = 310 Holstein Friesian crossbred cattle) and 139 smallholder dairy farms in Bengaluru Rural District of Karnataka, involving a total of 307 Holstein Friesian crossbred cattle. The study was approved by the Institutional Animal Ethical Committee of ICAR-National Dairy Research Institute, Southern Regional Station, Bengaluru, Karnataka, India (Approval number: CPCSEA/IAEC/LA/SRS-ICAR-NDRI-2021/No.011).

On-site surveys were conducted at the farms between October 2021 and November 2022, employing a meticulously designed questionnaire. An extensive literature review was conducted to identify key factors for the questionnaire on lameness prevalence and risk factors. Subject matter expert consultations aided in refining questionnaire components. Structured to cover herd composition and management practices, each question offered closed responses for ease of data collection. A pilot study with twenty farms ensured validity and prompted necessary question modifications. The refined questionnaire was re-administered to validate its reliability, resulting in an effective data-gathering tool. The questionnaire utilized in this study is provided as a supplementary file. Informed consent was obtained from all owners of the dairy farms for their participation in the research study.

### Assessment of animal-based risk factors

Animal-based factors were recorded based on a literature review, encompassing cow age, parity, body weight (calculated using Shaeffer’s formula [29]), milk yield, and stage of lactation. Body Condition Score (BCS) was assessed visually on a scale from 1 (lean) to 5 (fat) as per [30], categorizing cattle with a score of ≤ 2 as emaciated, 3 as normal, and 4 or more as obese. Animal hygiene was evaluated for legs, udders, and flank regions on a scale of 1–4, with scores 1 and 2 indicating cleanliness and 3 and 4 denoting dirtiness [31, 32].

Injuries to the left and right hock and knee joints were assessed on a scale of 0–3, where scores 0 and 1 indicated healthy joints and scores 2 and 3 denoted injured joints [33]. Before inspection, hooves were cleaned, and a thorough examination was conducted, identifying and recording hoof lesions using the International Committee for Animal Recording (ICAR) claw health atlas as a reference [34]. The presence or absence of lesions was documented.

### Assessment of lameness in animals

Lameness scoring involved assessing the animal’s gait both standing and walking, using a 5-point scale (Table 1) [35]. After milking, cattle walked on even, flat surfaces for 10–15 m, while their walking was recorded using a Nikon DX – D5100 digital camera positioned approximately 8 to 10 m away on a tripod stand. Two independent experts analyzed the videos to ascertain the degree of lameness, categorizing cattle with scores of 1 and 2 as non-lame and those with scores ≥ 3 as lame. The inter-observer agreement among the experts for commercial and smallholder dairy farms was evaluated using Kappa statistic (κ).

**Table 1:** Lameness Scoring System used in the study to determine the prevalence of lameness [35]

Locomotion Score	Interpretation	Description of Locomotion
1	Normal	Normal walk with a flat back
2	Mild lameness	Normal walk but with an arched back
3	Moderate lameness	Slight abnormal walk, short stride with one or more legs
4	Lameness	Visibly lame, but able to bear some weight on all legs
5	Severe lameness	Almost complete transfer of weight from an affected leg

### Assessment of management-based risk factors

Management factors were meticulously recorded, encompassing details such as the type of housing, flooring types in sheds and yards, and the presence or absence of bedding, all assessed through visual inspection. The cleanliness levels of sheds were determined by estimating the percentage of the floor covered by dung in the lying areas, providing a floor cleanliness score on a scale of 0–3 (Score 0, clean: ≤0.5 cm or a film of manure on the floor; 1, bit dirty: ≤1 cm or a fine layer of manure; 2, dirty; 1–3 cm manure thickness; 3, very dirty: >3 cm manure thickness). Additionally, other relevant factors were documented, including the provision of hoof trimming and footbath facilities, which was determined through direct inquiry with farm owners.

Furthermore, the study gathered information on access to pasture grazing and yards, along with the duration of such access. This comprehensive approach to data collection aimed to capture a holistic understanding of the various management practices employed on dairy farms, contributing to a thorough assessment of their potential impact on the prevalence of lameness in the studied cattle populations.

### Statistical analysis

The analysis employed basic descriptive statistics to calculate the median for age, body weight, parity, and milk yield, as well as the percentage of different variables such as lameness score, body condition score (BCS), animal hygiene, and hock and knee injuries. The association between potential risk factors and lameness was initially explored using a bivariate model, specifically the chi-square test.

To assess the contribution of potential risk factors in predicting the occurrence of lameness (binary response), and to determine adjusted odds ratios (OR) with a 95% confidence interval for subgroups of risk factors, a binary logistic regression model was applied. Variables deemed significant (*P* < 0.05) in the bivariate model were selected as candidates for inclusion in the logistic regression analysis. Notably, among age and parity, only parity was included in the regression analysis due to its practical applicability in Indian dairy farming conditions and to prevent collinearity issues.

To address the high dimensionality of the data and capture the underlying structure of the explanatory variables, principal components analysis (PCA) was performed [36]. PCA was applied to all explanatory independent variables (risk factors) using a correlation matrix. Categorical variables were transformed into numeric values through optimal scaling in PCA. The number of principal components was determined by examining the scree plots of PCA with different component numbers.

The entire analysis was conducted using SPSS version 22 software package (IBM Corp., Armonk, NY, USA), and the statistical significance level was set at 0.05 for all analyses. This comprehensive approach ensured a thorough exploration of potential risk factors and their association with the prevalence of lameness in the studied dairy cattle populations.

## Results

### Animal and management characteristics in commercial and smallholder dairy farms

In both commercial and smallholder dairy farming contexts, a comprehensive analysis of key demographic and production metrics was conducted. The median age of cows in these herds was found to be five years, with a first quartile (Q1) of four years and a third quartile (Q3) of six years, resulting in an Inter Quartile Range (IQR) of two years. Notably, the median body weight of cows in commercial dairy farms was recorded at 532.03 Kg, whereas in smallholder dairy farms, it stood at 378.73 Kg.

Parity, an important determinant of reproductive history, exhibited variations between commercial and smallholder setups. In commercial farms, the median parity was two, with Q1 and Q3 values of two and four, respectively, yielding an IQR of two. Conversely, in smallholder farms, the median parity was slightly higher at three, with Q1 and Q3 values of two and three, respectively, resulting in an IQR of one.

The primary metric of milk yield, crucial for assessing productivity, was observed to have a consistent median of 11 L/d across all herds. However, the range extended from 7.5 to 15.3 L/d, with an IQR of 7.8 L/d, highlighting the inherent variability in individual cow performance.

Additional detailed animal-based parameters for both commercial and smallholder dairy farms were presented in Table 2, providing further insights into the multifaceted nature of dairy production systems. This comprehensive dataset serves as a valuable resource for understanding and optimizing dairy farming practices. The inter-observer agreement for scoring lameness in dairy cows on commercial farms was determined to be 0.76, indicating substantial agreement strength. Conversely, for smallholder dairy farms, the inter-observer agreement was calculated at 0.59, indicating a moderate level of agreement among the experts in assessing lameness.

**Table 2:** Percentage of crossbred cows in each category (number) of animal-based parameters in commercial (*n* = 310) and smallholder dairy farms (*n* = 307)

Risk factors	Score 0	Score 1	Score 2	Score 3	Score 4	Score 5
Lameness score (scale 1–5)		42.58 (132) + 62.54 (192)	35.48 (110) + 32.89 (101)	12.90 (40) + 3.91 (12)	5.16 (16) + 0.65 (2)	3.87 (12) + 0
Body Condition Score (BCS) (scale 1–5)		2.90 (9) + 0	13.54 (42) + 20 (62)	53.87 (167) + 66.45 (206)	23.22 (72) + 11.61 (36)	6.45 (20) + 0.98 (3)
Animal hygiene score (Scale 1–4)		72.90 (226) + 58.30(179)	21.61 (67) + 27.69 (85)	4.83 (15) + 13.35 (41)	0.64 (2) + 0.65 (2)	
Hock injury (Scale 0–3)	64.51 (200) + 48.2 (148)	24.51 (76) + 40.06 (123)	9.68 (30) + 11.07 (34)	1.29 (4) + 0.65 (2)		
Knee injury (Scale 0–3)	59.57 (185) + 49.83 (153)	22.58 (70) + 36.8 (113)	17.1 (53) + 13.35 (41)	0.64 (2) + 0		

The prevalence of housing systems in commercial and smallholder dairy farms was investigated, revealing distinct patterns in management practices. Loose housing systems were dominant in 70.3% of animals within commercial dairy farms, whereas smallholder dairy farms predominantly tethered their animals, either within sheds, yards, or under trees, particularly during daytime hours. Flooring materials varied across both farm types, with stone slab floors being the most prevalent (51.21%, *n* = 316), followed closely by concrete (46.35%, *n* = 286), and a smaller proportion utilizing earth (*n* = 15). Notably, approximately 70.18% of animal sheds across all farms lacked bedding provision. Cleaning frequencies also diverged between commercial and smallholder farms, with commercial farms typically conducting cleaning activities thrice daily (67.4%, *n* = 209), while smallholder farms tended to clean sheds once daily (53.4%, *n* = 164). Common herd management practices in commercial farms included routine hoof trimming (60.6%, *n* = 188) and footbath usage (54.5%, *n* = 169). Moreover, a subset of commercial farms implemented grazing routines, with four out of six farms allowing grazing periods of 3–4 h during the early morning. A minority of farmers (11.1%, *n* = 34) permitted extended grazing durations of 4–6 h in community grazing lands. These findings shed light on the diverse husbandry strategies employed across different dairy farming contexts.

### Prevalence of lameness in dairy cattle

The prevalence of lameness in HF crossbred dairy cattle was 13.69% (95% CI: 8.17–19.21) at the farm level and 13.29% (95% CI: 10.6–15.9) at the animal level. The study revealed an overall lameness prevalence of 21.9% in commercial dairy farms and 4.6% in smallholder dairy farms. Among the 617 examined crossbred cows, 82 (13.29%) were clinically lame, with lameness scores ranging from 3 to 5 (3–8.43%, *n* = 52; 4–2.92%, *n* = 18; 5–1.94%, *n* = 12). The majority (52.51%, *n* = 324) of cows were not lame (score 1), while 34.2% (*n* = 211) exhibited mild/subclinical lameness (score 2). All cows belonged to the Holstein–Zebu cross genotype, which was predominant in the area.

### Risk factors associated with lameness

Chi-square values demonstrated that age, body weight, parity, body condition score (BCS), hock and knee injury, hoof trimming, and the presence of hoof lesions were significantly (*P* < 0.05) associated with lameness in dairy cows of commercial farms (Table 3). In smallholder dairy farms, significant associations (*P* < 0.05) with the prevalence of lameness were observed for age, body weight, parity, milk yield, BCS, animal hygiene, flooring, and provision of bedding (Table 4).

**Table 3:** Bivariate analysis of nine risk factors for lameness in HF crossbred dairy cows of commercial dairy farms

Risk factors		Non-Lame	Lame	χ^2^	*p*-value
Age (years)	< 4	50 (94.3)	3 (5.7)	10.063	0.007
	4–5	135 (75.4)	44 (24.6)		
	> 6	57 (73.1)	21 (26.9)		
Bwt (kg)	< 456	61 (74.4)	21 (25.6)	8.840	0.012
	456–560	114 (74.0)	40 (26.0)		
	> 560	67 (90.5)	7 (9.5)		
Parity	< 2	55 (93.2)	4 (6.8)	16.651	0.000
	2–4	158 (77.8)	45 (22.2)		
	> 4	29 (60.4)	19 (39.6)		
Stage of lactation	Early (0–90 d)	64 (70.3)	27 (29.7)	4.629	0.099
	Mid (91–180 d)	35 (83.3)	7 (16.7)		
	Late (> 181 d)	143 (80.8)	34 (19.2)		
BCS	Normal	144 (89.4)	17(10.6)	44.058	0.000
	Low	30 (48.4)	32 (51.6)		
	High	68 (78.2)	19 (21.8)		
Hock injury	Healthy	219 (81.1)	51 (18.9)	11.342	0.001
	Injured	23 (57.5)	17 (42.5)		
Knee injury	Healthy	207 (81.5)	47 (18.5)	9.669	0.002
	Injured	35 (62.5)	21 (37.5)		
Hoof trimming	Yes	155 (82.4)	33 (17.6)	5.357	0.021
	No	87 (71.3)	35 (28.7)		
Hoof lesions	Absent	209 (82.6)	44 (17.4)	16.593	0.000
	Present	33 (57.9)	24 (42.1)		

Bwt, Body weight; BCS, Body Condition Score. P value < 0.05 is considered as statistically significant. Figures in parenthesis under non-lame and lame are percentages of animals

**Table 4:** Bivariate analysis of risk factors for lameness in HF crossbred dairy cows of smallholder dairy farms

Risk factors		Non-Lame	Lame	χ ^2^	*p*-value
Age (years)	< 4	61 (98.4)	1 (1.6)	4.714	0.095
	4–5	211 (95.5)	10 (4.5)		
	> 6	21 (87.5)	3 (12.5)		
Bwt (kg)	< 456	219 (96.9)	7 (3.1)	5.791	0.055
	456–560	70 (92.1)	6 (7.9)		
	> 560	4 (80.0)	1 (20.0)		
Parity	< 2	61 (98.4)	1 (1.6)	15.362	0.000
	2–4	212 (96.4)	8 (3.6)		
	> 4	20 (80.0)	5 (20.0)		
Milk yield (kg)	< 7	36 (94.7)	2 (5.3)	8.390	0.015
	7–16	214 (97.3)	6 (2.7)		
	> 16	43 (87.8)	6 (12.2)		
BCS	Normal	194 (97.0)	6(3)	6.612	0.037
	Low	61 (89.7)	7 (10.3)		
	High	38 (97.4)	1 (2.6)		
Animal Hygiene	Clean	256 (97.0)	8 (3.0)	10.137	0.001
	Dirty	37 (86.0)	6 (14.0)		
Flooring	Earthen	12 (3.9)	3 (20.0)	9.559	0.008
	Concrete	26 (100)	0 (0)		
	Stone slab	255 (95.9)	11 (4.1)		
Bedding	Yes	76 (91.6)	7 (8.4)	3.921	0.048
	No	217 (96.9)	7 (3.1)		
Hoof lesions	Absent	245 (96.5)	9 (3.5)	3.496	0.062
	Present	48 (90.6)	5 (9.4)		

Bwt, Body weight; BCS, Body Condition Score. P value < 0.05 is considered as statistically significant. Figures in parenthesis under non-lame and lame are percentages of animals

The binary logistic regression model applied to commercial dairy farms identified several risk factors for lameness. These included larger body weight (> 560 kg; *P* = 0.01), low and high BCS (< 3 score; *P* = 0.01 and 4 and above score; *P* = 0.001), higher parity (4 and above; *P* = 0.08), stone slab flooring (*P* = 0.001), absence of hoof trimming (*P* = 0.04), and the presence of hoof lesions (*P* = 0.002) (Table 5).

**Table 5:** Binary logistic regression of lameness with risk factors in commercial dairy farms (*n* = 310)

Risk factors		Coefficient	Odds Ratio	Confidence Interval	*p*-Value
Constant		-1.864	0.155		0.017
Body weight (Kg)	< 456 (Ref*)	-	-	-	0.038
	456–560	0.932	2.540	0.770–8.377	0.126
	> 560	1.281	3.599	1.345–9.631	0.011
Parity	< 2 (Ref*)	-	-	-	0.001
	2–4	-2.671	0.069	0.017–0.289	0.000
	> 4	-0.827	0.437	0.173–1.108	0.081
Body Condition Score (BCS)	Normal (score3) (Ref*)	-	-	-	0.000
	Thin (< 3 score)	-0.982	0.375	0.166–0.845	0.018
	Obese (4 and above)	1.434	4.193	1.736–10.131	0.001
Floor-type		2.182	8.862	2.582–30.411	0.001
Absence of hoof trimming		-1.182	0.442	0.197–0.991	0.048
Presence of hoof lesions		-1.864	0.307	0.147–0.638	0.002

Ref*, Reference category. P value < 0.05 is statistically significant from the reference category

In smallholder dairy farms, lameness exhibited a significant positive association with increasing parity of the animal (OR = 0.14, CI = 0.04–0.55), larger body weight of the cow (OR = 0.14, CI = 0.01–1.97), stone slab floor type (OR = 7.403, CI = 1.54–35.48), and a positive association with the dirty cow (OR = 0.15, CI = 0.04–0.54). These findings provide valuable insights into the specific risk factors influencing lameness in dairy cows across different farm types, aiding in the development of targeted prevention and management strategies.

### Principal components of the risk factors associated with lameness

In both commercial and smallholder dairy farms, the Kaiser-Meyer-Olkin Measure of Sampling Adequacy was 0.633 and 0.531, respectively, for various risk factors. The overall significance of the correlation matrix was tested using Bartlett’s test of sphericity for risk factors, and it was significant at the 1% level, indicating the suitability of data for factor analysis (PCA) using risk factors associated with lameness in dairy cattle.

**Fig. 1:**
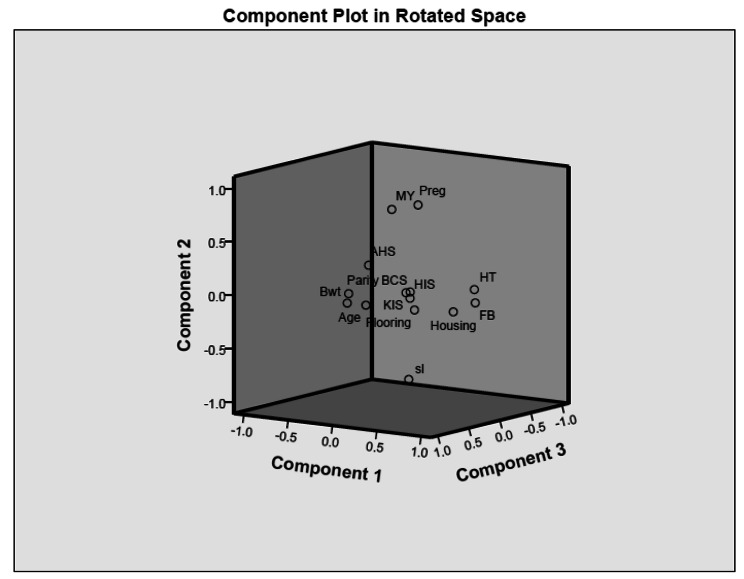
Component plot in rotated space for risk factors of lameness in commercial dairy farms. (Bwt: body weight; BCS: Body Condition Score; AHS: Animal hygiene score; HIS: Hock injury score; KIS: Knee injury score; MY: Milk yield; Preg: Pregnancy status of the animal; HT: hoof trimming; FB: Footbath; sl: stage of lactation)

In the PCA, five main components emerged from the animal and management-based risk factors associated with lameness, each having eigenvalues greater than 1. These components explained 71.32% of the total variance in commercial farms and 61.21% of the total variance in smallholder dairy farms (Table 6). The extracted component matrix for commercial farms (Table 7; Fig. 1) showed that the first principal component was represented by significant positive high loadings of management-based factors, while the second component explained high loadings for the parity of the animal. The third component explained the body condition of the animal. The fourth and fifth components accounted for higher loading for hock and knee injury and production efficiency of an animal, respectively.

**Table 6:** Total variance explained by various risk factors in commercial farms

Component	Initial Eigenvalues			Rotation Sums of Squared Loadings		
	Total	% of Variance	Cumulative %	Total	% of Variance	Cumulative %
1	3.780	27.003	27.003	3.015	21.538	21.538
2	2.088	14.913	41.916	2.054	14.672	36.210
3	1.586	11.325	53.241	1.991	14.222	50.433
4	1.382	9.872	63.113	1.719	12.277	62.710
5	1.150	8.212	71.325	1.206	8.615	71.325
6	0.889	6.352	77.677			
7	0.712	5.086	82.764			
8	0.631	4.504	87.268			
9	0.502	3.588	90.856			
10	0.444	3.170	94.027			
11	0.354	2.531	96.557			
12	0.265	1.890	98.447			
13	0.169	1.208	99.656			
14	0.048	0.344	100.000			

Extraction Method: Principal Component Analysis

**Table 7:** Extracted principal component matrix for various risk factors in dairy cattle from commercial farms

Risk factors	Component^a^				
	1	2	3	4	5
Footbath	0.884	− 0.323		0.104	− 0.187
Hoof trimming	0.756	− 0.342	0.184	0.224	
Housing type	0.691	− 0.271	0.191	− 0.154	− 0.273
Age	0.639	0.504	0.136	− 0.402	
Body weight	− 0.609	0.300	0.405	− 0.124	− 0.112
Flooring	0.602		− 0.233	− 0.450	− 0.126
Hock injury	0.555	0.414		0.493	
Stage of lactation	− 0.130	0.203		0.209	− 0.820
Pregnancy status	0.233	− 0.252			0.750
Milk yield			0.183		0.728
Animal hygiene		0.324	− 0.159	0.106	0.345
Parity	0.525	0.606	0.136	− 0.408	0.172
Knee injury	0.393	0.424	− 0.235	0.607	0.106
Body condition			0.835	0.273	− 0.116

Extraction Method: Principal Component Analysis. ^a^Five components extracted

These findings suggest that these components effectively capture the underlying structure and relationships among the various risk factors associated with lameness in dairy cattle, providing a more streamlined and interpretable representation of the complex interplay of factors influencing lameness prevalence in both commercial and smallholder dairy farms.

## Discussion

The present study reported a lameness prevalence of 21.9% in commercial dairy farms and 4.6% in smallholder dairy farms. Globally, the mean estimate for the prevalence of lameness in dairy cows is documented as 22.8%, with a wide range of herd prevalence from 0 to 88% [12]. However, studies on lameness prevalence in Indian dairy cows are limited and often confined to individual farms, revealing prevalence levels ranging from 8.1 to 30.5% [13–17].

The observed prevalence of lameness in commercial farms aligns closely with previous reports on crossbred cows in various regions of India [37–39]. Interestingly, the relatively low prevalence of lameness in smallholder dairy farms may be attributed to the attentive care and management of crossbred cows at the individual farm level, where only 2–3 cows are typically kept by each farmer. However, variations in lameness prevalence rates could also be influenced by diverse housing and management conditions [20, 40]. These findings underscore the importance of individualized farm-level care and management practices in influencing the prevalence of lameness in dairy cows.

The bivariate analysis conducted in both commercial and smallholder dairy farms revealed that age, body weight, parity of the cow, BCS, and hoof lesions are significantly correlated factors with the prevalence of lameness. Additionally, in commercial farms, the stage of lactation, hock and knee joint injuries, and hoof trimming practices, and in smallholder farms, milk yield of the animal, cow hygiene, flooring, and bedding were also found to be significantly correlated factors with the prevalence of lameness. These findings align with previous research by [20], who identified five major risk factors, including BCS, presence of claw overgrowth, days in milk, herd size, and parity, as significant contributors to lameness in a meta-analysis study. Similarly, in smallholder dairy farms [19], reported six out of 13 risk factors, including parity and BCS, as associated risk factors for lameness in bivariate analysis, consistent with the observations in this study. The results of the binary logistic model further confirmed that larger body weight, higher parity, low BCS, hard flooring like stone slab, dirty animal, and the presence of hoof lesions were significant predictors of lameness. These comprehensive analyses shed light on the multifactorial nature of lameness in dairy cows and emphasize the importance of considering a range of factors for effective prevention and management strategies.

The finding that higher parity increases a cow’s risk of lameness is consistent with previous studies [20, 40]. In both commercial and smallholder dairy farms, the present study observed a significant (*p* < 0.01) impact of parity on the risk of lameness, particularly for cows in parity > 4. Similarly [20], reported that cows in parities 4 and higher have 2.46 times increased odds of being diagnosed as lame compared to first lactation animals. Multiparous cows may experience a cumulative effect of calving-associated stress, metabolic changes throughout parities, and housing-related deficiencies due to the longer time spent in the confined artificial environment. These factors could be detrimental to hoof conformation, claw health, locomotion, and exacerbate existing problems [24, 41–43]. The evidence suggests that addressing the specific needs and challenges faced by multiparous cows is crucial for effective lameness prevention and management in dairy herds.

The strong association between low BCS and lameness in the current study aligns with the findings of previous researchers (17, 20, 40]. A low BCS in cows is both phenotypically and genetically positively associated with susceptibility to lameness [41; 44]. Lameness can result in reduced movement, including slower feeding rates and decreased feed intake, all of which have the potential to contribute to a decline in the body condition of cows [44, 45]. The decreased movement is partially attributed to a reduced digital cushion, a fatty pad located in the claw capsule that serves as a shock absorber when the third phalanx bears the weight of the cow during the interaction of the hoof with the flooring [45, 46]. It is hypothesized that during periods of excessive weight loss due to reduced feed intake, fat is mobilized from the digital cushion, diminishing its force-dissipating capacities. Consequently, cows may experience impaired mobility as the decreasing dimensions of the digital cushion lead to increased pressures on the corium, germinative epithelium, and distal phalanx, promoting the development of further traumatic claw lesions [44–48]. These insights underscore the importance of maintaining optimal body condition in cows as part of lameness prevention and management strategies in dairy farming.

The present study demonstrated that the odds of lameness increased in dirty animals within smallholder dairy farms according to the logistic regression model. This finding is consistent with previous research indicating that dirty conditions predispose cows to lameness [40, 49]. Poor hygiene, characterized by the accumulation of dung and urine in lying areas and passages, can lead to various hoof lesions, ultimately resulting in lameness [17, 24] [50]. similarly observed associations, reporting that cows with dirty and very dirty leg hygiene scores had approximately 3 and 10 times increased odds of being lame. The flooring of the shed also plays a role in claw health and influences the occurrence of hoof lesions leading to lameness in dairy cows. Binary logistic regression analysis in both commercial and smallholder dairy farms revealed that cows reared on stone slab floors had significantly higher odds of being lame (*p* < 0.05). These results align with previous reports suggesting that the hardness, abrasiveness, and slipperiness features of concrete floors contribute to foot lesions and lameness [19, 51, 52]. The findings emphasize the importance of maintaining clean conditions and appropriate flooring to mitigate the risk of lameness in dairy cows.

In commercial farms, the bivariate analysis revealed a significant (*p* < 0.05) correlation between lameness and hock and knee injuries. Similar associations were observed in studies by [17, 40, 49]. It was found that lame cows tend to lie down for longer periods, increasing their exposure to the lying surface and potentially putting them at risk of developing hock and knee lesions [19, 51].

Conversely, the existence of hock lesions may cause gait abnormalities due to mechanical restrictions of joint flexion, infections at the lesion site, or pain related to the lesion (40; 50]. Sometimes, the type of flooring surfaces can contribute to the occurrence of hock joint ulcerations and carpal joint injuries, acting as risk factors for lameness [17, 50, 51]. These findings underscore the intricate relationship between lameness and injuries, emphasizing the importance of understanding and addressing factors such as lying behaviour, flooring conditions, and the presence of lesions in effective lameness prevention strategies.

Approximately 90% of the causes of lameness involve hoof lesions [22, 53, 54]. Hoof lesions are considered significant indicators and risk factors for lameness in dairy cows [55]. We also observed that the odds of lameness in crossbred cows increase with the presence of hoof lesions. Similarly [19], found that cows with hoof lesions had seventeen times higher chances of becoming lame than those with normal hooves. Lameness is a complex issue influenced by various metabolic factors, including housing and management conditions that require prolonged standing over hard surfaces. Natural weight-bearing forces contribute to mechanical overloading of claws, leading to the development of hoof disorders, ultimately resulting in lameness [56]. Understanding and addressing hoof lesions are crucial components of comprehensive lameness prevention and management strategies in dairy farming.

The high correlation among most of the risk factors, along with high Kaiser-Meyer-Olkin (KMO) values for a measure of sample adequacy and significant chi-square values for Bartlett’s test of sphericity, confirms the suitability of risk factors associated with lameness for multivariate data analysis, specifically principal component analysis in dairy cattle. The results of the principal component analysis suggest that the extracted components can be effectively used to substantially reduce the number of recorded risk factors while explaining the maximum variability in the prevalence of lameness in dairy cattle.

The significant positive high loadings of the first component emphasize the importance of farm management factors, highlighting the significance of proper and comfortable housing, provision of bedding, hoof trimming, and footbath in lameness prevention. The second and third components account for body structure and body condition, suggesting that dairy cows could be successfully selected at an optimum age or parity with better body condition. The fourth component extracted can identify the importance of body lesions in affecting the productivity of the animal, while the fifth component accounts for production efficiency based on milk yield. This multivariate approach aids in simplifying the understanding of the complex interactions among various risk factors associated with lameness in dairy cattle.

In this cross-sectional study, the authors employed convenience sampling to assess the prevalence of lameness. Data were gathered directly from farm owners to explore the correlation between lameness in dairy cows and various animal and management-related risk factors. This sampling method was chosen due to its practicality, reflecting factors such as geographical proximity, availability, and willingness to participate. However, a notable limitation of the study lies in its approach to sampling from smallholder and commercial farms. While a similar number of animals were selected from each farm type, the representation of farms was not proportionately balanced due to time constraints. Given the significant impact of herd size and management practices on lameness, this imbalance in farm representation may have introduced bias into the results, potentially undermining the accuracy and generalizability of the findings. Further, the moderate level of agreement observed among assessors in scoring lameness on smallholder dairy farms may have been due to the limited sample size. This could be improved by implementing standardized assessment protocols, and enhanced communication among stakeholders is essential for minimizing bias and improving the accuracy of lameness assessments in smallholder dairy farming contexts.

Future research should aim to not only increase the number of animals sampled but also ensure a balanced representation of diverse farm types. This approach will enhance the study’s ability to capture the wide spectrum of agricultural practices and facilitate a comprehensive understanding of lameness prevalence. Additionally, forthcoming studies should delve into diverse management interventions aimed at preventing lameness in Indian dairy cattle, thereby offering tailored strategies for effective prevention. Longitudinal research is paramount for assessing the sustained impact and long-term viability of these interventions on lameness prevalence and overall herd health. Furthermore, investigating the economic implications and cost-effectiveness of these strategies will yield valuable insights for dairy farmers and industry stakeholders, aiding in informed decision-making and resource allocation.

## Conclusion

The study reveals variations in the prevalence of lameness between commercial and smallholder dairy production systems, with crossbred cows in smallholder farms exhibiting a lower prevalence compared to those in commercial farms. Key risk factors associated with lameness in crossbred dairy cattle include age, parity, body weight of the animal, body condition score, cleanliness, flooring type, hock and knee injuries, and the presence of hoof lesions. The findings emphasize the importance of comprehensive management practices in addressing both animal and housing-related factors to mitigate the risk of lameness in both large and small dairy herds. Proper attention to factors such as hygiene, flooring conditions, and regular hoof care is crucial for the overall well-being and productivity of dairy cattle, contributing to the strategic management of lameness in diverse dairy farming settings. Implementing regular hoof trimming programmes, facilitated through training or professional services, is imperative for addressing lameness in both smallholder and commercial dairy farms, thereby enhancing cow welfare, productivity, and overall hoof health.

### Electronic supplementary material

Below is the link to the electronic supplementary material.

Supplementary Material 1

## Data Availability

The datasets generated during and analyzed during the current study are available from the corresponding author on reasonable request.

## References

[1] Flower FC, Weary DM (2006). Effect of hoof pathologies on subjective assessments of dairy cow gait. J Dairy Sci.

[2] Huxley JN (2013). Impact of lameness and claw lesions in cows on health and production. Livest Sci.

[3] Dolecheck K, Bewley J (2018). Animal board invited review: dairy cow lameness expenditures, losses and total cost. Anim.

[4] Robcis R, Ferchiou A, Berrada M, Ndiaye Y, Herman N, Lhermie G, Raboisson D (2023). Cost of lameness in dairy herds: an integrated bioeconomic modeling approach. J Dairy Sci.

[5] Shearer JK, Hutjens MF, Endres MI (2017). Managing the herd to minimize lameness. Large dairy Herd Management.

[6] Whay HR, Shearer JK (2017). The impact of lameness on welfare of the dairy cow. Vet Clin North Am Food Anim.

[7] Browne N, Hudson CD, Crossley RE, Sugrue K, Kennedy E, Huxley JN, Conneely M (2022). Lameness prevalence and management practices on Irish pasture-based dairy farms. Ir Vet J.

[8] Jensen KC, Oehm AW, Campe A, Stock A, Woudstra S, Feist M, Müller KE, Hoedemaker M, Merle R (2022). German farmers’ awareness of lameness in their dairy herds. Front Vet Sci.

[9] Salfer JA, Siewert JM, Endres MI (2018). Housing, management characteristics, and factors associated with lameness, hock lesion, and hygiene of lactating dairy cattle on Upper Midwest United States dairy farms using automatic milking systems. J Dairy Sci.

[10] Randall LV, Green MJ, Green LE, Chagunda MGG, Mason C, Archer SC, Huxley JN (2018). The contribution of previous lameness events and body condition score to the occurrence of lameness in dairy herds: a study of 2 herds. J Dairy Sci.

[11] Costa JH, Burnett TA, von Keyserlingk MA, Hötzel MJ (2018). Prevalence of lameness and leg lesions of lactating dairy cows housed in southern Brazil: effects of housing systems. J Dairy Sci.

[12] Thomsen PT, Shearer JK, Houe H (2023). Prevalence of lameness in dairy cows. Vet J.

[13] Singh S (1998). Incidence of lameness in dairy cows and buffaloes in Punjab State. Indian Vet J.

[14] Sood P, Nanda AS (2006). Effect of lameness on estrous behavior in crossbred cows. Theriogenology.

[15] Chakrabarti A, Kumar P (2016). Incidences of foot diseases of cattle in Bihar, India. Int J Agric Sci Res.

[16] Kumar R, Kataktalware MA, Senani S, Sivaram M, Ramesha KP (2019). Risk factors associated with incidence of hoof disorders in cross bred dairy cattle under field conditions. Int J Curr Microbiol Appl Sci.

[17] Sharma A, Phillips CJ (2019). Lameness in sheltered cows and its association with cow and shelter attributes. Animals.

[18] Gupta RK, Lathwal SS, Ruhil AP, Dash SK, Singh M (2016). Effect of non-genetic factors on incidence of lameness in Karan Fries cross bred cows. Indian J Anim Sci.

[19] Kumar R, Kataktalware MA, Senani S, Sivaram M, Devi GL, Niketha L, Ramesha KP (2021). Risk factors Associated with the lameness in crossbred dairy cattle maintained under field conditions. J Anim Res.

[20] Oehm AW, Knubben-Schweizer G, Rieger A, Stoll A, Hartnack S (2019). A systematic review and meta-analyses of risk factors associated with lameness in dairy cows. BMC Vet Res.

[21] Adams AE, Lombard JE, Fossler CP, Román-Muñiz IN, Kopral C (2017). Associations between housing and management practices and the prevalence of lameness, hock lesions, and thin cows on US dairy operations. J Dairy Sci.

[22] Sadiq MB, Ramanoon SZ, Mossadeq WS, Mansor R, Syed-Hussain SS (2020). Cow-and herd-level factors associated with lameness in dairy farms in Peninsular Malaysia. Prev Vet Med.

[23] Zanon T, Alrhmoun M, Katzenberger K, Poulopoulou I, Gauly M (2023). Identifying housing and management factors associated with lameness in small-scaled mountain dairy farms with different housing systems. Livest Sci.

[24] Oehm AW, Merle R, Tautenhahn A, Jensen KC, Mueller KE, Feist M, Zablotski Y (2022). Identifying cow–level factors and farm characteristics associated with locomotion scores in dairy cows using cumulative link mixed models. PLoS ONE.

[25] Hernandez-Mendo O, Von Keyserlingk MAG, Veira DM, Weary DM (2007). Effects of pasture on lameness in dairy cows. J Dairy Sci.

[26] Blowey RW (2020). Cattle lameness and hoofcare: an illustrated guide.

[27] Bell N, Bacon D, Craven E, Crowe S, Newsome R, Oikonomou G, Pedersen S, Reader J, Wilson J (2022). Dairy cattle lameness: a roundtable discussion. Livest.

[28] Garvey M (2022). Lameness in dairy cow herds: Disease Aetiology, Prevention and Management. Dairy.

[29] Johnson PW. Livestock weights from measurements. Principles and Practices of Dairy Farm Management; 1940.

[30] Ferguson JD, Azzaro G, Licitra G (2006). Body condition assessment using digital images. J Dairy Sci.

[31] Schreiner DA, Ruegg PL (2003). Relationship between udder and leg hygiene scores and subclinical mastitis. J Dairy Sci.

[32] Cook NB. (2006) Footbath alternatives. University of Wisconsin Madison, United States of America. Available online: http://www.vetmed.wisc.edu/dms/fapm/fapmtools/6lame/Footbath_Alternatives. pdf. Accessed: 23 January 2023.

[33] Gibbons J, Vasseur E, Rushen J, De Passillé AM (2012). A training programme to ensure high repeatability of injury scoring of dairy cows. Anim Welf.

[34] Egger-Danner C, Nielsen P, Fiedler A, Müller K, Fjeldaas T, Döpfer D, Daniel V, Bergsten C, Cramer G, Christen AM, Stock KF, Cole JB. (2015) ICAR Claw Health Atlas. ICAR Technical Series, 18: 45.

[35] Sprecher DEA, Hostetler DE, Kaneene JB (1997). A lameness scoring system that uses posture and gait to predict dairy cattle reproductive performance. Theriogenology.

[36] Massy WF (1965). Principal components regression in exploratory statistical research. J Am Stat Assoc.

[37] Nandi S, Roy S, Mukherjee P, Goswami A, Majumder D (2008). Epidemiology of lameness in dairy cattle of hilly region of west Bengal: the influence of pain on performance. Livest Res Rural Dev.

[38] Parmar JJ, Patel PB, Kag BG (2014). Incidence of hoof affections in cattle of North Gujarat. Indian J Vet Surg.

[39] Baranwal A, Gaur GK, Pruthviraj DR (2019). Prevalence of lameness in crossbred and Tharparkar cattle: a comparison. J Entomol Zool Stud.

[40] Solano L, Barkema HW, Pajor EA, Mason S, LeBlanc SJ, Heyerhoff JZ, Orsel K (2015). Prevalence of lameness and associated risk factors in Canadian holstein-friesian cows housed in freestall barns. J Dairy Sci.

[41] Bicalho RC, Machado VS, Caixeta LS (2009). Lameness in dairy cattle: a debilitating disease or a disease of debilitated cattle? A cross-sectional study of lameness prevalence and thickness of the digital cushion. J Dairy Sci.

[42] Foditsch C, Oikonomou G, Machado VS, Bicalho ML, Ganda EK, Lima SF, Rossi R, Ribeiro BL, Kussler A, Bicalho RC (2016). Lameness prevalence and risk factors in large dairy farms in upstate New York. Model development for the prediction of claw horn disruption lesions. PLoS ONE.

[43] Newsome R, Green MJ, Bell NJ, Chagunda MGG, Mason CS, Rutland CS, Sturrock CJ, Whay HR, Huxley JN (2016). Linking bone development on the caudal aspect of the distal phalanx with lameness during life. J Dairy Sci.

[44] Randall LV, Green MJ, Chagunda MGG, Mason C, Archer SC, Green LE, Huxley JN (2015). Low body condition predisposes cattle to lameness: an 8-year study of one dairy herd. J Dairy Sci.

[45] Green LE, Huxley JN, Banks C, Green MJ (2014). Temporal associations between low body condition, lameness and milk yield in a UK dairy herd. Prev Vet Med.

[46] Räber M, Lischer CJ, Geyer H, Ossent P (2004). The bovine digital cushion–a descriptive anatomical study. Vet J.

[47] Räber M, Scheeder MR, Ossent P, Lischer CJ, Geyer H (2006). The content and composition of lipids in the digital cushion of the bovine claw with respect to age and location–a preliminary report. Vet J.

[48] Newsome RF, Green MJ, Bell NJ, Bollard NJ, Mason CS, Whay HR, Huxley JN (2017). A prospective cohort study of digital cushion and corium thickness. Part 1: associations with body condition, lesion incidence, and proximity to calving. J Dairy Sci.

[49] Oehm AW, Jensen KC, Tautenhahn A, Mueller KE, Feist M, Merle R (2020). Factors associated with lameness in tie stall housed dairy cows in South Germany. Front Vet Sci.

[50] Sadiq MB, Ramanoon SZ, Mansor R, Syed-Hussain SS, Shaik Mossadeq WM (2017). Prevalence of lameness, claw lesions, and associated risk factors in dairy farms in Selangor, Malaysia. Trop Anim Health Prod.

[51] Brenninkmeyer C, Dippel S, Brinkmann J, March S, Winckler C, Knierim U (2013). Hock lesion epidemiology in cubicle housed dairy cows across two breeds, farming systems and countries. Prev Vet Med.

[52] Mishra M, Upadhyay D, Gurav A, Domple V (2017). Effect of floor on lameness in crossbred dairy cow. Int J Livest Res.

[53] Solano L, Barkema HW, Mason S, Pajor EA, LeBlanc SJ, Orsel K (2016). Prevalence and distribution of foot lesions in dairy cattle in Alberta. Can J Dairy Sci.

[54] O’Connor AH, Bokkers EA, de Boer IJ, Hogeveen H, Sayers R, Byrne N, Ruelle E, Shalloo L (2019). Associating cow characteristics with mobility scores in pasture-based dairy cows. J Dairy Sci.

[55] Manske T, Hultgren J, Bergsten C (2002). Prevalence and interrelationships of hoof lesions and lameness in Swedish dairy cows. Prev Vet Med.

[56] Shearer JK, Van Amstel SR, Brodersen BW (2012). Clinical diagnosis of foot and leg lameness in cattle. Vet Clin North Am Food Anim.

