# Supplementary material for: Assessing lameness prevalence and associated risk factors in crossbred dairy cows across diverse management environments
Source: BMC Vet Res. 2024 May 25;20:229. doi: 10.1186/s12917-024-04093-w (PMC11127402; doi:10.1186/s12917-024-04093-w)
Supplement: Supplementary file 1 — Supplementary Material 1 [file 12917_2024_4093_MOESM1_ESM.docx]

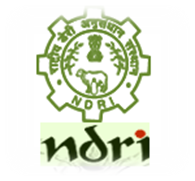

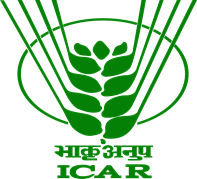
Dairy Production Section

Southern Regional Station,

ICAR-National Dairy Research Institute

(Deemed University)

Bengaluru- 560030 (Karnataka), India

Major Advisor- Dr. M. A. Kataktalware Scholar- Dr. Priyanka Patoliya

**QUESTIONNAIRE**

Serial no: _____________ Date: / / 2021

1. **General Information**
2. Name of the farmer: __________________________________________________
3. Address: ____________________________________________________
4. Village: ___________________ District: ___________________
5. Phone no: ___________________
6. **Herd Composition**

| S. no. | Category of animals | Breed: ______________ | Breed: ______________ | Breed: ______________ |
| --- | --- | --- | --- | --- |
| 1 | Calves Male  (0-6 month) Female |  |  |  |
| 2 | Calves Male  (6-12 month) Female |  |  |  |
| 3 | Heifer |  |  |  |
| 4 | Milking animals |  |  |  |
| 5 | Dry animals |  |  |  |
| 6 | Bull |  |  |  |
| 7 | Bullock |  |  |  |
|  | Total |  |  |  |

1. **Animal Managemental Practices**
2. **Animal Description**

| **Breed** |  |  |  |
| --- | --- | --- | --- |
| **Animal no.** |  |  |  |
| **Age (Years)** |  |  |  |
| **Parity** |  |  |  |
| **Stage of lactation** |  |  |  |
| **Milk yield (kg/day) of previous day** |  |  |  |
| **Date of last time AI done** |  |  |  |
| **Pregnancy status (P/NP)** |  |  |  |
| **Days to first heat (days)** |  |  |  |
| **Days open** |  |  |  |
| **No. of services per conception** |  |  |  |
| **Deworming** |  |  |  |
| **Vaccination** |  |  |  |

1. **Animal Body Weight Estimation**

| **Length of animal** |  |  |  |
| --- | --- | --- | --- |
| **Girth of animal** |  |  |  |
| **Body weight (kg)**  **G^2^*L/300** |  |  |  |

1. **Animal Housing Management**

| **Type of Housing** | Close/tied/stall |  |  |  |
| --- | --- | --- | --- | --- |
|  | Loose/free |  |  |  |
|  | Semi loose |  |  |  |
| **Roof** |  |  |  |  |
| **Floor type** | Kutcha |  |  |  |
|  | Bricked |  |  |  |
|  | Stone slab |  |  |  |
|  | Concrete |  |  |  |
|  | Any other |  |  |  |
| **Bedding** | YES/NO |  |  |  |
| **Bedding type** (if yes)  Rubber mat/straw/any other | |  |  |  |
| **Drainage** | PRESENT/ABSENT |  |  |  |

1. **Animal Feeding Management**

| **Feeding** | Stall fed |  |  |  |
| --- | --- | --- | --- | --- |
|  | Grazing |  |  |  |
|  | If grazing- duration (hours) |  |  |  |
|  | Timing of grazing (morning/evening) |  |  |  |
| **Green fodder** | Type/Variety |  |  |  |
|  | Frequency |  |  |  |
|  | Quantity (kg/animal/day) |  |  |  |
| **Dry fodder** | Type |  |  |  |
|  | Frequency |  |  |  |
|  | Quantity (kg/animal/day) |  |  |  |
| **Concentrate** | Type/Brand |  |  |  |
|  | Composition  (Grains/Brans/Cake/Others) |  |  |  |
|  | Frequency |  |  |  |
|  | Quantity (kg/animal/day) |  |  |  |
| **Mineral mixture** | Brand Composition and bioavailability |  |  |  |
|  | Frequency (regular/irregular) |  |  |  |
|  | Quantity (kg/animal/day) |  |  |  |
| **Fresh and clean drinking water** | Source |  |  |  |
|  | Method of offering |  |  |  |
|  | Frequency (ad.lib /once/twice) |  |  |  |
|  | Quantity (kg/animal/day) |  |  |  |

1. **Hygiene Management**

| **House cleaning** (Frequency) |  |  |  |
| --- | --- | --- | --- |
| **Animal cleaning** (Frequency) |  |  |  |
| **Animal hygiene scoring (1, 2, 3 and 4)** |  |  |  |
| **Farm cleanliness score (0, 1, 2 and 3)** |  |  |  |

1. **Herd Health Management**

| **Health problem (Tick all that apply and/or insert other health issues)** | **Ranking on effort to identify the problem (1 = greatest)** | **Ranking on effort put into controlling the problem (1 = greatest)** | **Ranking on cost to business**  **(1 = greatest)** |
| --- | --- | --- | --- |
| **Lameness** |  |  |  |
| **Mastitis** |  |  |  |
| **Fertility** |  |  |  |
| **Other disease**  **(Specify: …….….….)** |  |  |  |
| **Other disease**  **(Specify: ….…….….)** |  |  |  |

1. **Body Condition Scoring (BCS)**

| **Score (1, 2, 3, 4 and 5)** |  |  |  |
| --- | --- | --- | --- |

1. **Animal Lameness/Gait Scoring**

| **Score (1, 2, 3, 4 and 5)** |  |  |  |
| --- | --- | --- | --- |
|  |  |  |  |

1. **Hoof Health Assessment**

| **Claw traits** | Claw length |  |  |  |
| --- | --- | --- | --- | --- |
|  | Claw height |  |  |  |
|  | Lateral Claw width |  |  |  |
|  | Medial Claw width |  |  |  |
|  | Heel height |  |  |  |
| **Hock injury scoring (0, 1, 2 and 3)** | Left |  |  |  |
|  | Right |  |  |  |
| **Knee injury scoring (0, 1, 2 and 3)** | Left |  |  |  |
|  | Right |  |  |  |
| **Hoof trimming** | YES/NO |  |  |  |
| **If yes** | Who is doing that job? (farmer/specialist hoof trimmer/veterinarian/any other person) |  |  |  |
|  | Frequency/Timing |  |  |  |
| **Foot bath** | YES/NO |  |  |  |
| **If yes** | Type of footbath solution |  |  |  |
|  | Frequency/Timing |  |  |  |
|  | When they did change of solution |  |  |  |

1. **Condition of Hoof (Present/Absent)**

| **Overgrown hoof/claw** |  |  |  |
| --- | --- | --- | --- |
| **Corkscrew claw** |  |  |  |
| **Sole hemorrhages** |  |  |  |
| **White line disease** |  |  |  |
| **Sole ulcer** |  |  |  |
| **Heel erosion** |  |  |  |
| **Digital dermatitis** |  |  |  |
| **Hoof cracks** |  |  |  |
| **Vertical fissure** |  |  |  |
| **Horizontal fissure** |  |  |  |
| **Digital Dermatitis** |  |  |  |
| **Interdigital dermatitis** |  |  |  |
| **Interdigital hyperplasia** |  |  |  |
| **Others**  **(Specify: ………...)** |  |  |  |
| **Others**  **(Specify: ….….….)** |  |  |  |
